# Using Synthetically Engineered Guide RNAs to Enhance CRISPR Genome Editing Systems in Mammalian Cells

**DOI:** 10.3389/fgeed.2020.617910

**Published:** 2021-01-28

**Authors:** Daniel Allen, Michael Rosenberg, Ayal Hendel

**Affiliations:** Institute of Nanotechnology and Advanced Materials, The Mina and Everard Goodman Faculty of Life Sciences, Bar-Ilan University, Ramat-Gan, Israel

**Keywords:** CRISPR-Cas9, engineered nuclease, gRNA, chemical modifications, genome editing, gene therapy, CRISPR therapeutics

## Abstract

CRISPR-Cas9 is quickly revolutionizing the way we approach gene therapy. CRISPR-Cas9 is a complexed, two-component system using a short guide RNA (gRNA) sequence to direct the Cas9 endonuclease to the target site. Modifying the gRNA independent of the Cas9 protein confers ease and flexibility to improve the CRISPR-Cas9 system as a genome-editing tool. gRNAs have been engineered to improve the CRISPR system's overall stability, specificity, safety, and versatility. gRNAs have been modified to increase their stability to guard against nuclease degradation, thereby enhancing their efficiency. Additionally, guide specificity has been improved by limiting off-target editing. Synthetic gRNA has been shown to ameliorate inflammatory signaling caused by the CRISPR system, thereby limiting immunogenicity and toxicity in edited mammalian cells. Furthermore, through conjugation with exogenous donor DNA, engineered gRNAs have been shown to improve homology-directed repair (HDR) efficiency by ensuring donor proximity to the edited site. Lastly, synthetic gRNAs attached to fluorescent labels have been developed to enable highly specific nuclear staining and imaging, enabling mechanistic studies of chromosomal dynamics and genomic mapping. Continued work on chemical modification and optimization of synthetic gRNAs will undoubtedly lead to clinical and therapeutic benefits and, ultimately, routinely performed CRISPR-based therapies.

## Introduction

Up until the discovery of the Clustered Regularly Interspaced Short Palindromic Repeats (CRISPR) system, genome editing was limited in its capabilities. CRISPR is simpler and more versatile than other genome editing tools, such as zinc-finger nucleases (ZFNs) and Transcription-Activator-Like-Effector-Nucleases (Porteus and Carroll, 2005; Carroll, 2011, 2014). The CRISPR system components are modified from the prokaryotic adaptive immune system. Throughout evolution, bacteria and archaea acquired the ability to store copies of portions of invading foreign genetic material such as plasmids, phage genomes, or RNA, as segments between clustered repetitive sequences in the genome. These sequences are transcribed together into CRISPR RNAs (crRNAs), which are subsequently utilized to recognize and destroy the invading complementary DNA or RNA molecules by Cas nucleases (Horvath and Barrangou, 2010; Terns and Terns, 2011; Morange, 2015). The current nomenclature identifies two classes of the CRISPR-Cas systems, Class 1 and 2 (Makarova et al., 2020). Class 2 is distinguished by a multi-domain effector Cas nuclease and uses trans-activating CRISPR RNA (tracrRNA), in addition to crRNA, for target recognition and cleavage (Makarova et al., 2020). With three types in each class and more than a dozen subtypes, the CRISPR-Cas system represents a fruitful field for developing bioengineering tools.

Since it was first reported in 2013 that the CRISPR system could be repurposed into a reliable and straightforward genome editing technique in mammalian cells (Cong et al., 2013; Mali et al., 2013; Hsu et al., 2014), the CRISPR-Cas system has championed the field of gene editing. The most popular tool developed based on the CRISPR-Cas system is CRISPR-Cas9 (Jiang and Doudna, 2017), derived from *Streptococcus pyogenes*. Cas9 belongs to the Class 2 type II system and is a multi-domain endonuclease that requires both crRNA and tracRNA to introduce a double-strand break (DSB) at the target genomic site. After crRNA and tracrRNA anneal together to form a guide RNA (gRNA), they assemble a ribonucleoprotein (RNP) complex with a Cas9 molecule to direct site-specific DNA cleavage. The complex then scans the DNA for a complementary sequence to the 20 nucleotides on its 5′ end, termed the guide region (spacer region), with an adjacent upstream protospacer adjacent motif (PAM) sequence (5′-NGG-3′ in *S. pyogenes*) (Jiang and Doudna, 2017). Once the PAM is recognized, the guide region of the gRNA undergoes seed nucleation to form an A-form-like helical RNA:DNA hybrid duplex. Only once the RNA and DNA complete R-loop formation, also known as the zipped conformation, and structural rearrangement of the nuclease domains commence, can the endonuclease cut the DNA creating a DSB (Jiang et al., 2015; Jiang and Doudna, 2017). One of the benefits of the two-component system is that the gRNA can be modified independently from the Cas nuclease, making the alteration of CRISPR as a genome-editing tool easy and flexible with almost unlimited target capability and high efficiency (Hsu et al., 2014; Moon et al., 2019). The guide can be adapted to the target by switching the 20 nucleotides with any sequence complementary to a desired target site in the genome (providing the genomic sequence is flanked by a PAM sequence). In addition to Cas9 (Type II), other members of the Class 2 system have also been exploited for targeted editing, including Cas12a (formally Cpf1), that belongs to Type V, and Cas13a (Type VI). In contrast to Cas9, Cas12a utilizes a single molecule gRNA with a 3′ oriented spacer region and a 5′ pseudoknot (5′ handle). Additionally, Cas12a nuclease cleavage produces cohesive double-strand breaks (DSBs) (compared to the predominantly blunt-end DSB created by Cas9) and relies on different PAM recognition sequences. Similar to Cas12a, Cas13a utilizes a single-molecule gRNA with a 3′ oriented spacer region; however, in contrast to Cas12a, it targets complementary RNA sequences instead of DNA (Chylinski et al., 2014; Shmakov et al., 2017; Tang and Fu, 2018). Together, these CRISPR-Cas formulations confer a convenient technology for researchers to conduct sequence-specific editing of nucleic acids in a wide variety of cell types and experimental set-ups.

Due to CRISPR's wide-ranging applications, as well as its relative simplicity and highly flexible nature, it has been catapulted to the forefront of research in a remarkably vast number of organisms, from bacteria to humans (Wang et al., 2013; Guo and Li, 2015; Sid and Schusser, 2018; Xue et al., 2018; Yao et al., 2018; Ge et al., 2019; Munoz et al., 2019; Song et al., 2019; Soni, 2020). The CRISPR system can be utilized to knock-out genes by creating a DSB at the site of interest in the genome. Following the CRISPR-induced DSB, the endogenous cellular DNA repair mechanism, called non-homologous end joining (NHEJ), can repair the break, often resulting in small insertions or deletions (indels), which can lead to frameshift mutations, thereby inactivating the target gene (Yang et al., 2020). Hence, measuring the extent of indels on the site of interest, following CRISPR-mediated editing, is considered a gold standard for assessing the CRISPR activity in cultured cells and *in vivo*. Researchers also have used the CRISPR system to knock-in specific genes by taking advantage of the homology-directed repair (HDR) pathway (Yang et al., 2020), where the cell uses a template to repair the DSB. Naturally, the cell can use the sister chromatid or the homologous chromosome as a template for HDR; however, researchers have shown the ability to use an exogenous donor template to introduce genes into the CRISPR cut site (Porteus, 2016).

One of the main challenges facing researchers since the beginning of the CRISPR era is how to optimize the CRISPR system for translation to clinical therapies (Zhang, 2020). One promising direction in which CRISPR-based gene editing is currently being exploited is *ex vivo* gene therapy using cells of hematopoietic origin. In this procedure, hematopoietic stem and progenitor cells (HSPCs) or T lymphocytes are isolated from the patient's blood, undergo the desired gene correction *ex vivo*, and are then transfused back to the patient's bloodstream. Disorders that can be treated by this method include β-globin-associated diseases such as sickle-cell anemia and β-thalassemia (Dever et al., 2016; DeWitt et al., 2016; Park et al., 2019; Romero et al., 2019; Wu et al., 2019), as well as Severe Combined Immunodeficiency (SCID) (Pavel-Dinu et al., 2019), Polyendocrinopathy Enteropathy X-linked Syndrome (IPEX) (Goodwin et al., 2020), Wiskott-Aldrich Syndrome (Rai et al., 2020), X-linked chronic granulomatous disease (De Ravin et al., 2017), and Mucopolysaccharidosis Type 1 (Gomez-Ospina et al., 2019). Furthermore, T lymphocytes can be engineered using CRISPR to recognize and attack tumor cells (Gao et al., 2019; Stadtmauer et al., 2020). However, since the majority of genetic diseases and tumors occur in tissues that cannot be conveniently isolated and edited *ex vivo*, other therapeutic options must be explored. One such direction that is pursued using CRISPR-based genome editing is *in vivo* delivery of the editing complexes to the target tissues, with a focus on more accessible tissues such as the eye, liver, muscle, and cervix (Hirakawa et al., 2020). This could potentially lead to treatments for a number of diseases including cervical cancer (Zhen and Li, 2017), an inherited form of blindness Leber congenital amaurosis type 10 (LCA10) (Maeder et al., 2019), among others. Albeit, the application of the CRISPR-Cas9 system for clinical purposes still faces significant obstacles. First and foremost, safety is a critical parameter. The popular method for CRISPR-mediated gene editing in cultured cells involves transfection with plasmid DNA that expresses both gRNA and Cas9 protein under constitutive promoters (Ran et al., 2013). However, the plasmid system is problematic for use in clinical applications since plasmid DNA, as well as any foreign DNA, can trigger an innate intracellular immune response, especially in primary cells (Sun et al., 2013). Unregulated constitutive expression of integrated CRISPR-Cas9 can also destabilize the genome through persistent DSB generation. Therefore, for clinical purposes, the CRISPR-Cas9 system must possess a limited intracellular lifespan to allow for quick and efficient gene editing while minimizing off-target effects. To that end, clinically relevant CRISPR-Cas9 systems must be developed that would avoid triggering the innate immune response and increase specificity in primary cells. The current solution to these issues is to use formulations of gRNAs together with Cas9 mRNA or protein instead of plasmid DNA. Together, these drawbacks have garnered a tremendous concerted effort from researchers to modify the CRISPR-Cas9 system to improve its editing capabilities as well as its ability to be tolerated in human cells. Although equally as much work has been done to modify the Cas9 protein to improve on its characteristics, herein, we discuss the chemical modifications that have been used specifically on the gRNA to adapt this bacterial element to a more effective, accurate, and versatile genome-editing tool while concurrently attempting to improve safety in order to achieve therapeutic relevance.

## Production of gRNAs

**Figure 1:**
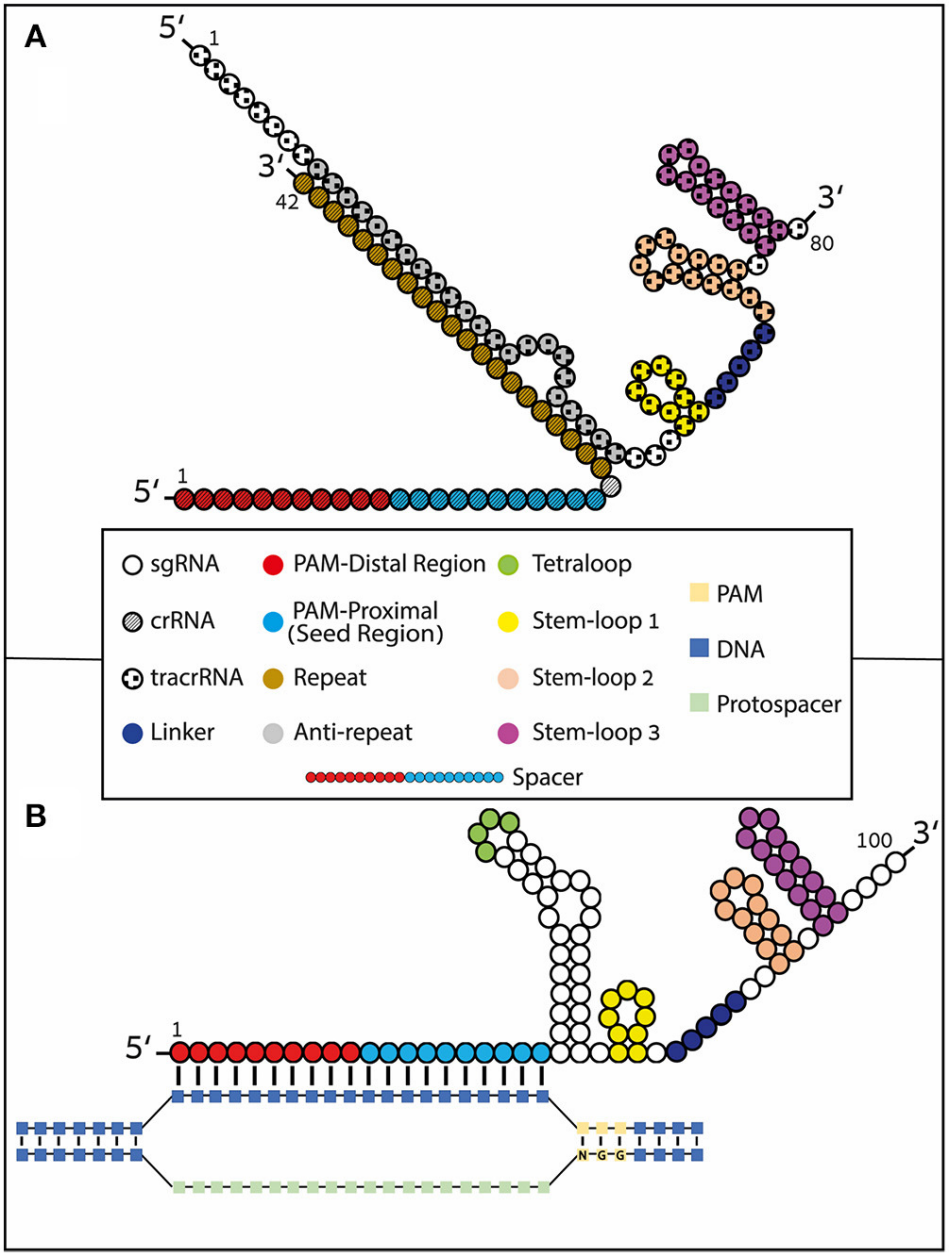
Type II CRISPR formulations. gRNAs contain 4 loop structures: tetraloop (green), Stem-loop 1 (yellow), Stem-loop 2 (orange), and Stem-loop 3 (magenta). Stem-loop 2 and tetraloop do not interact with Cas9 as they protrude from the nuclease (Konermann et al., 2015). The spacer region of the guide undergoes Watson and Crick base pairing with the complimentary stand to the DNA protospacer. The spacer region (also known as guide region) is typically 20 nucleotides long but it has been shown that it can be shortened or lengthened (to include hairpin structures) at the 5′ end. The spacer region can be divided into two regions: the PAM-proximal (seed) region and the PAM-distal region. **(A)** Naturally occurring crRNA [~42 nt (striped nucleotides)] containing the DNA-binding spacer sequence and the trans-activating tracrRNA [80 nt (Rahdar et al., 2015) (checkered nucleotides)] annealed together through Watson and Crick base-pairing by the repeat (brown) and anti-repeat (gray) regions. **(B)** Synthetic sgRNA formulation where the crRNA and tracrRNA are covalently fused by a tetraloop. R-loop formation is depicted with Watson and Crick base pairing of the RNA:DNA heteroduplex.

Like other types of RNA, gRNAs consist of ribonucleotides covalently bound together by phosphodiester bonds. To be able to complex with the Cas protein, gRNAs can come in one of two basic formulations: a two-part molecule or a single-guide molecule (sgRNA). In nature, gRNA is found as a two-part molecule consisting of crRNA (~36–42 nt), which contains the DNA-binding spacer sequence, and the tracrRNA (~67–89 nt) (Jinek et al., 2012). The crRNA sequence can be divided into a guide region and a repeat region, while the tracrRNA sequence consists of an anti-repeat region and three stem-loop (numbered 1–3) structures. The guide region forms the gRNA:DNA heteroduplex through Watson and Crick base pairing with the DNA target site, while the repeat region and the anti-repeat region form the repeat:anti-repeat duplex also through Watson and Crick base pairing (Jinek et al., 2012; Nishimasu et al., 2014) (Figure 1A). The second type of gRNA that can complex with Cas is a synthetic sgRNA (~100 nt) where the bridged portion between the crRNA and the tracrRNA is covalently linked by an artificial tetraloop (Jinek et al., 2012) (Figure 1B). The synthetic sgRNA system has been shown to achieve equivalent or higher efficiency compared to the two-part RNA system (Kelley et al., 2016; Shapiro et al., 2020).

There are a few conventional ways to produce gRNAs (Moon et al., 2019), including chemical synthesis using oligonucleotide synthesizers, *in vitro* transcription (IVT), and intracellular production via gRNA-expressing DNA vectors which hijack the host cell's transcription machinery. However, since primary cells are known to mount an innate immune response to the foreign DNA (Sun et al., 2013), as well as to the *in vitro* transcribed gRNAs (as discussed below), chemical synthesis represents a cost-effective, expeditious alternative that produces highly purified gRNA at scalable quantities. Due to the short length of the gRNA, chemical synthesis allows for the swift and uncomplicated formational changes as well as the addition of different moieties. Recently, Taemaitree and colleagues presented a simplified method for producing sgRNAs via synthesis of the variable guide sequence (20 nt) and subsequently ligating the product to the remaining constant region (79 nt) by a triazole linkage (Taemaitree et al., 2019). Together, these advancements in the engineering of synthetically modified gRNA have enabled tremendous improvements in CRISPR-mediated genome editing's stability, specificity, and safety. These improvements have also expanded the applications of CRISPR-Cas9, such as techniques for enhanced HDR and improved genome imaging tools.

## Increasing CRISPR Efficiency Through Stabilization of the gRNA

In order to use CRISPR-Cas9 genome editing in a therapeutic setting, the first problem that needs to be addressed is gRNA stability. RNA is highly unstable compared to DNA and is extremely vulnerable to both endo- and exo-nucleases. The many years of progress in enhancing small RNA-based technologies, such as antisense RNA and RNA interference (RNAi) (Levin, 2019), includes improving RNA stability by incorporating chemical modifications onto the small RNAs (Braasch et al., 2003; Chiu and Rana, 2003; Behlke, 2008; Bennett and Swayze, 2010; Deleavey and Damha, 2012; Lennox and Behlke, 2020). Likewise, a pioneering study by Hendel et al. demonstrated that for optimal gRNA efficiency, the guide must be modified in a way that protects it from degradation by RNA nucleases. This can be achieved by chemically modifying the gRNA ends to reduce degradation by exonucleases, thus improving the guide's stability (Hendel et al., 2015a). Modifications can be made both on the ribose ring as well as on the phosphodiester bond to reduce nuclease susceptibility. Research has also shown that the order in which the gRNA and Cas9 are delivered can change gRNA stability, as the Cas9 itself seems to confer the gRNA some level of protection from degradation when delivered as an RNP complex. However, the major contribution of Hendel et al. was proof that chemically modified gRNAs work efficiently in concert with Cas9 mRNA or protein in primary cells, which do not tolerate the introduction of plasmid DNA. The ability to chemically modify gRNAs opened the door for the development of more efficient and safer gene-editing methods that can be appropriate for clinical applications in primary cells. Nonetheless, caution should be exercised when introducing RNA modifications since further analysis found that over modification of the gRNA in the seed region, the ten nucleotides in the spacer region that recognize the target DNA closest to the PAM sequence, also known as the PAM-proximal portion, inhibits proper DNA:RNA hybridization and can significantly hinder efficiency (Rahdar et al., 2015; Basila et al., 2017; Yin et al., 2017). Another possible side effect of gRNA modification can be increased cytotoxicity, leading to cellular death, a major problem many researchers are actively seeking to solve (Basila et al., 2017). Several studies have shown that gRNA modifications in Type V CRISPRs (Cas12a), including 3′ terminal chemical modifications (Li et al., 2017; McMahon et al., 2018) and crRNA elongation (Bin Moon et al., 2018; Park et al., 2018), stabilize the complex and enhance editing efficiency. Additionally, in Cas12a, modifications in the seed region or on the 5′ handle were not well-tolerated (Safari et al., 2019). New formulations of Cas9-gRNA complexes with various RNA modifications are continually being developed to achieve the proper balance between benefits and side effects. Below we review the types of chemical modifications and their impact on various aspects of CRISPR-Cas9 applications *in vitro* and *in vivo* (Table 1).

**Table 1:** gRNA modifications to improve CRISPR-Cas9 efficiency in cultured mammalian cells.

**Modification(s)**	**Modification location**	**Effect on genome editing efficiency**	**References**
M	Terminal residues	↑^#^	Hendel et al., 2015a; Rahdar et al., 2015
MS	Terminal residues	↑^#^	Hendel et al., 2015a; Basila et al., 2017; Finn et al., 2018
	Spacer (PAM-distal region)	↑^*^	Yin et al., 2017; Finn et al., 2018; Mir et al., 2018
	Spacer (tracrRNA-binding region)	↑^*^	Yin et al., 2017; Finn et al., 2018; Mir et al., 2018
	Spacer (Seed region)	↓	Yin et al., 2017; Mir et al., 2018
MSP	Terminal residues	↑^#^	Hendel et al., 2015a
cEt	Spacer (PAM-distal region)	↑	Rahdar et al., 2015
	Spacer (tracrRNA-binding region)	↑	Rahdar et al., 2015
	Spacer (Seed region)	↓	Rahdar et al., 2015
2′-F	Spacer (PAM-distal region)	↑	Rahdar et al., 2015
	Spacer (tracrRNA-gbinding region)	↑	Rahdar et al., 2015
	Spacer (Seed region)	↓	Rahdar et al., 2015; O'Reilly et al., 2019
2′-F + PS	Spacer (PAM-distal region)	↑	Yin et al., 2017; Mir et al., 2018
	Spacer (tracrRNA-binding region)	↑	Yin et al., 2017; Mir et al., 2018
	Spacer (Seed region)	↓	Yin et al., 2017; Mir et al., 2018
	Spacer (Seed region, Cas9-non-interacting residues)	↑^*^	Yin et al., 2017; Mir et al., 2018
PS	Whole crRNA	↑	Rahdar et al., 2015

* *additionally validated in vivo*.

# *additionally validated in human primary cells*.

*2′-O-methyl (M or 2′-O-Me); 2′-O-methyl 3′phosphorothioate (MS); 2′-O-methyl-3′-thioPACE (MSP); S-constrained ethyl (cEt); 2′-fluoro (2′-F); and phosphorothioate (PS)*.

### Chemical Modifications on gRNA Termini

**Figure 2:**
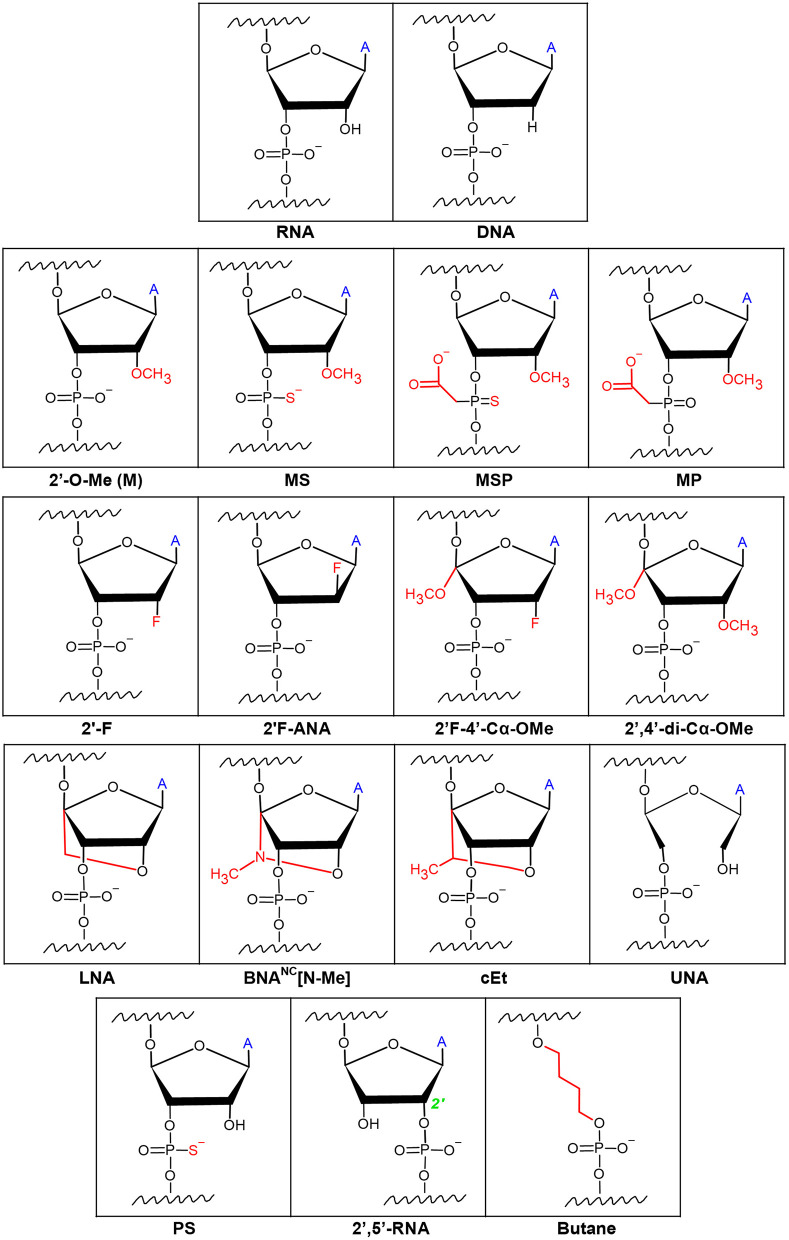
Chemical modifications on the ribose rings and phosphate backbone of gRNAs. Ribose modifications are typically placed at the 2′OH as it is readily available for manipulation. Simple modifications at the 2′OH include 2′-O-Me, 2′-F, and 2′F-ANA. More extensive ribose modifications such as 2′F-4′-Cα-OMe and 2′,4′-di-Cα-OMe combine modification at both the 2′ and 4′ carbons. Phosphodiester modifications include sulfide-based Phosphorothioate (PS) or acetate-based phosphonoacetate alterations. Combinations of the ribose and phosphodiester modifications have given way to formulations such as 2′-O-methyl 3′phosphorothioate (MS), or 2′-O-methyl-3′-thioPACE (MSP), and 2′-O-methyl-3′-phosphonoacetate (MP) RNAs. Locked and unlocked nucleotides such as locked nucleic acid (LNA), bridged nucleic acids (BNA), S-constrained ethyl (cEt), and unlocked nucleic acid (UNA) are examples of sterically hindered nucleotide modifications. Modifications to make a phosphodiester bond between the 2′ and 5′ carbons (2′,5′-RNA) of adjacent RNAs as well as a butane 4-carbon chain link between adjacent RNAs have been described. ‘A’ symbolizes the nitrogen base of the RNA.

As mentioned above, a significant issue with gRNAs is their marked tendency to be degraded by exonucleases. Hendel et al. showed that sgRNAs with three different independent chemical modifications at both termini increased editing efficacy by protecting the exposed ends from degradation (Hendel et al., 2015a). Chemical modifications comprising of 2′-O-methyl (M or 2′-O-Me), 2′-O-methyl 3′phosphorothioate (MS), or 2′-O-methyl-3′-thioPACE (MSP) (Figure 2) were incorporated at three terminal nucleotides at both the 5′ and 3′ ends of individual sgRNAs. These modifications, specifically MS and MSP, substantially increased stability, resulting in a high level of indels at the on-target site compared to the indel frequencies obtained with the unmodified sgRNA. Moreover, with few exceptions, the increase in the on-target activity was accompanied by only a minor effect on off-target activity, thus achieving favorable on-target:off-target ratios. This was the first time it was shown that sgRNA chemical modifications enhance intracellular stability, thereby increasing genome editing efficacy when Cas9 and sgRNAs are co-delivered into human primary cells (Hendel et al., 2015a). A later study by Basila et al. systematically evaluated several combinations of MS end modifications in both the two-part system and sgRNA as well as two types of intracellular delivery mechanisms for the editing complexes: electroporation and cationic lipid transfection (Basila et al., 2017). The cationic lipid delivery technique previously suggested that liposomes protect gRNA molecules from RNase degradation in the cytosol or culture medium (Anderson et al., 2015; Liang et al., 2015). Basila et al. demonstrated that one MS modification at the 5′ and 3′ ends of the sgRNA molecule, or two MS modifications at the 5′ end of the crRNA and 3′ end of the tracrRNA were enough to improve editing efficiency when electroporated with Cas9 mRNA into K562 cells (Basila et al., 2017). However, when electroporated as an RNP complex, these modifications did not significantly increase editing efficiency. They also observed only a small increase in editing efficiency when gRNAs were delivered together with Cas9 mRNA into HeLa or U2OS cell lines, while the number and placement of modifications on gRNA termini showed a significant effect on cellular toxicity (Basila et al., 2017). Taken together, the mode of intracellular delivery of gRNA-Cas9 complexes, whether gRNA is delivered with Cas9 mRNA or protein, and the number and positions of chemical modifications are all key factors that must be considered when planning CRISPR-Cas9 gene editing experiments. Recently, a thoroughly optimized protocol for using end-modified sgRNA in human primary HSPCs was evaluated, demonstrating high editing efficiency and specificity through the delivery of the CRISPR system as an RNP complex (Shapiro et al., 2020, 2021). This method can potentially be adapted for therapeutic purposes in other hematopoietic cells such as T and B lymphocytes, and Natural Killer (NK) cells.

### Extensive and Complete Chemical Modification of gRNA Backbone

Adding modifications only on the 3′ and 5′ ends of gRNAs would protect the gRNA from exonucleolytic but not endonucleolytic activity inside the cells, which also may impair the editing efficiency by reducing gRNA stability. To address this, a study by Rahdar et al. focused on modifying the crRNA while expressing tracrRNA and Cas9 separately from plasmid DNA in HEK293T cells (Rahdar et al., 2015). They demonstrated that using a phosphorothioate (PS) (Figure 2) modified backbone in tandem with 2′-O-Me modifications on the terminal five nucleotides on both ends of the crRNA enhanced the editing activity, presumably by diminishing crRNA susceptibility to nucleolytic cleavage. In addition, adding modifications known to increase RNA affinity to DNA, such as 2′-fluoro (2′-F) and S-constrained ethyl (cEt) (Figure 2), on the crRNA inside of the PAM-distal and tracrRNA-binding regions, respectively, further increased editing activity. On the contrary, any modifications on the 2′ carbon in the ribose ring were not tolerated in the PAM-proximal (seed) region, presumably since the seed region is critical for target DNA recognition by Cas9 (Jiang et al., 2015). Lastly, they noted that it is possible to shorten the crRNA down to 29 nucleotides and still maintain its efficiency (Rahdar et al., 2015). However, in this study, the tracrRNA remained unmodified, and the potential of using these chemical modifications *in vivo* was not explored. To address this, Finn et al. examined the impact of sgRNA modifications on genome editing efficiency in mouse and rat liver *in vivo* (Finn et al., 2018). They designed lipid nanoparticles containing Cas9 mRNA and sgRNA and discovered that 2′-O-Me and PS chemical modifications on both termini of sgRNA [similar to the MS used by Hendel et al. (2015a)], as well as on the internal residues in the crRNA and tracrRNA regions, resulted in more efficient *in vivo* genome editing compared to the unmodified sgRNA or sgRNA with only terminal modifications (Finn et al., 2018). Yin et al. also performed an extensive study of gRNA modifications in *in vivo* gene editing in mouse livers using lipid nanoparticles (Yin et al., 2017); however, they used the crystal structure of the CRISPR-Cas9 RNP complex to guide the optimization of combinations of sgRNA modifications. Previous work has shown that there are ~20 positions of nucleotides in both crRNA and tracrRNA that interact with the Cas9 protein via the 2′-OH group, and thus do not tolerate any 2′-OH modifications. To show the significance of maintaining these gRNA-Cas9 interactions, Yin et al. highlighted the complete abolishment of genome-editing capability when all 2′-OH sites were modified. By avoiding those 2′-OH sites, a sgRNA was designed with a pattern of PS, 2′-F, and 2′-O-Me modifications on the remaining non-Cas9-interacting nucleotides that maximized the editing efficiency both in HEK293 cells and in live animals. This underscored the importance of avoiding modifications on the endonuclease-interacting 2′-OH groups, maintaining the sgRNA-Cas9 hydrogen bonding, and modifying the other nucleotides to increase editing efficiency (Yin et al., 2017). Similar work was performed by Mir et al. where the modification pattern relied on the CRISPR-Cas9 complex crystal structure (Mir et al., 2018). Based on prior work in the field of RNA therapeutics, Mir et al. hypothesized that maximal 2′-modified ribose rings and modified backbone phosphate groups inside the crRNA and tracrRNA should generate the required gRNA formulation for clinical studies; albeit, all of the work in the study was conducted on HEK293 cells, without *in vivo* validation. They were able to obtain complete gRNA modification by combining the PS, 2′-F, and 2′-O-Me modifications which resulted in decreased Cas9 activity and as well as overall efficiency. Interestingly, they showed that the combination of heavily modified tracrRNA with completely modified crRNA exhibited satisfactory editing activity (Mir et al., 2018). An additional study by O'Reilly et al. utilized a broader variety of chemical modifications and linkers to test the compatibility and structure-activity relationships of engineered gRNAs with Cas9-mediated editing activity to try and lay out the foundation for a rational design of modified gRNAs (O'Reilly et al., 2019). The study focused solely on modifying crRNA while being mindful of the impact on the RNA's helix conformation. Modifications included: 2′F, 2′F-ANA, 2′,5′-RNA, 2′F-4′-Cα-OMe, 2′,4′-di-Cα-OMe, unlocked nucleic acids (UNA), locked nucleic acids (LNA), and butane linkers (Figure 2). The analysis of the relationship between these extensive modifications, the resulting structure of the RNA and RNP complex, and the subsequent intrinsic complex activity *in vitro* emphasized the necessity for maintaining an A-form-like helical structure of the crRNA in both the guide and the repeat regions. They also concluded that the guide region of crRNA, and especially the seed region, favor modifications that closely resemble the native RNA nucleotides, such as 2′-F, while more bulky modifications were less tolerable. Nevertheless, there was a clear discrepancy between the Cas9 activity in *in vitro* activity assays vs. in cultured cells after 2′-OH modification. Contrary to the *in vitro* activity assays, in cultured cells, any hydrogen-bond-disrupting modifications on the Cas9-interacting nucleotides reduced editing activity (O'Reilly et al., 2019). This highlighted the necessity for proper hydrogen bonding for Cas9-gRNA complexes in cultured cells. Therefore, when translating these discoveries to the clinic, the relevant modifications must be validated in primary cells and animal models.

## Increasing CRISPR Specificity by Limiting Off-target Editing

It is important to remember that CRISPR-Cas endonucleases did not naturally evolve to function as a highly specific gene-editing tool to edit mammalian genomes. In some cases, these bacterial nucleases have demonstrated significant off-target activity, leading to unintended DNA breaks at ectopic sites in the genome with only partial complementarity to the gRNA sequence (Li et al., 2019). While mutations or mismatches within the PAM sequence ostensibly abrogate Cas9 endonuclease activity (O'Geen et al., 2015a; Jiang and Doudna, 2017), mismatches within the guide region may be permitted (O'Geen et al., 2015b) resulting in the undesired cleavage of off-target DNA sequences. This creates a potential major pitfall for CRISPR-based therapies due to the well-understood correlation between increased DSBs to increased cellular toxicity and elevated immune response (Obe et al., 1992; Lips and Kaina, 2001; Nakad and Schumacher, 2016; Bednarski and Sleckman, 2019). Therefore, quantifying (Hendel et al., 2014, 2015b) and improving the accuracy, precision, and specificity of these nucleases (Tsai and Joung, 2016) is of major significance. Indeed, more accurate genome editing has been achieved via Cas9 nuclease modification itself (Kleinstiver et al., 2016; Slaymaker et al., 2016; Chen et al., 2017; Vakulskas et al., 2018). Additionally, Cas12a has been shown to be more specific than Cas9 at certain genomic sites (Kim et al., 2016) and may be more useful in particular settings. However, the orthogonal approach attempts to elevate CRISPR-Cas9 genome editing precision via chemical modifications on the gRNA, as discussed below (Table 2).

**Table 2:** gRNA modifications to improve CRISPR-Cas9 specificity in cultured mammalian cells.

**Modification(s)**	**Modification location**	**Effect on genome editing specificity**	**References**
Deoxyribonucleotide substitution	crRNA 3′	↑	Kartje et al., 2018
	Spacer (PAM-distal region)	↑	Yin et al., 2018
MP	Spacer (positions 5 and 11)	↑^#^	Ryan et al., 2018
LNA	Spacer (positions 10-14)	↑	Cromwell et al., 2018
BNA^NC^	Spacer (positions 10-14)	↑	Cromwell et al., 2018
tru-gRNA	5′ end of the spacer	↑	Fu et al., 2014
ggXX_20_ gRNA	5′ end of the spacer	↑	Cho et al., 2014

# *additionally validated in human primary cells*.

*2′-O-methyl-3′-phosphonoacetate (MP); locked nucleic acids (LNA); N-methyl substituted BNAs (2′,4′-BNA^NC^[N-Me]); truncated gRNA (tru-gRNA); and two added guanine residues on the 5′ end of the spacer sequence (ggXX_20_ gRNA)*.

### Chemical Modifications on Internal gRNA Residues

The aforementioned work by Yin et al. revealed that although PS, 2′-F, and 2′-O-Me modifications are tolerated in all of the non-Cas9 interacting nucleotides to improve gRNA stability, the extent of off-target editing between unmodified and modified sgRNA was comparable in both cultured cell lines and mice liver cells (Yin et al., 2017). Two independent studies systematically assessed the effect of modifying internal gRNA residues on Cas9 cleavage specificity. Ryan et al. sought to increase Cas9 cleavage specificity by altering the thermodynamic and kinetic properties of the gRNA-DNA heteroduplex formation, such as melting temperature (Ryan et al., 2018). They aimed to preserve sufficient duplex stability and relatively low dissociation rate on the fully complementary on-target genomic site while simultaneously decreasing the duplex stability and increasing the dissociation rate on the off-target sites with only partial gRNA complementarity. They first examined the on- and off-target editing by testing gRNA modifications 2′-O-Me, 2′-O-methyl-3′-phosphonoacetate (MP), MS, and MSP (Figure 2) in *in vitro* cleavage assays and then continued to assess the editing by NHEJ in cultured K562 cells, primary CD34^+^ HSPCs, and induced pluripotent stem cells. It was shown that MP modifications, incorporated at select sites in the ribose phosphate backbone of gRNAs (positions 5 and 11), along with modifications which protect the terminal positions (Hendel et al., 2015a), can reduce off-target cleavage activities while maintaining on-target cleavage editing (Ryan et al., 2018). Additionally, it has been shown that adding two types of bridged nucleic acids (BNAs), N-methyl substituted BNAs (2′,4′-BNA^NC^[N-Me]) and, to a lesser extent, locked nucleic acids (LNAs) (Figure 2), within the central portion of the guide region (positions 10–14) of crRNAs, considerably increases mismatch discrimination in the PAM-proximal and PAM-distal regions (Cromwell et al., 2018). Cromwell et al. conducted an extensive, high-throughput analysis of Cas9 cleavage specificity both *in vitro* and in cultured cells, combined with mechanistic studies to identify the precise stage during the Cas9-cleavage reaction that was affected by the BNA^NC^ and LNA substitutions (Cromwell et al., 2018). LNAs are conformationally restricted RNA nucleotides in which the 2′ oxygen on the ribose forms a covalent bond with the 4′ carbon (You et al., 2006). LNAs display improved base stacking and thermal stability compared to unmodified RNA, resulting in highly efficient binding to complementary nucleic acids and improved mismatch discrimination (You et al., 2006). BNA^NC^s are molecules with a six-membered bridged structure where the 2′ oxygen and the 4′ carbon are linked by a methyl-bound nitrogen. Even more effective than LNAs, BNA^NC^s can provide additional conformational flexibility for nucleic acid binding and greater nuclease resistance. In addition, BNA^NC^ nucleotides have been shown to be less toxic than LNA nucleotides when delivered to cultured cells (Manning et al., 2017). Both BNAs mentioned above improve specificity by inducing a more dynamic RNA-DNA duplex, thereby reducing the time the nuclease spends in the zipped conformation where cleavage is activated. The shorter interaction time in this conformation resulted in slower cleavage kinetics on the on-target sites but resulted in lowered Cas9-induced off-target DNA cleavage by several orders of magnitude (Cromwell et al., 2018), which on an overall scale was beneficial for the specificity of the genome editing.

### RNA Secondary Structures and Modified Spacer Length

There are at least five stages in the gRNA-mediated Cas9 cleavage reaction, most of which involve conformational changes both within the Cas9 protein and in the RNA-DNA helix (Lim et al., 2016). R-loop formation is particularly critical for the conformational change of Cas9, turning it into an active nuclease (Josephs et al., 2015; Sternberg et al., 2015). Since, as mentioned earlier, the chemical modifications that affect zipped conformation influence Cas9-gRNA complex off-target activity (Cromwell et al., 2018), it is plausible that manipulating the secondary structure or the length of the gRNA may improve genome editing precision as well. Accordingly, Fu et al. demonstrated that manipulating the spacer length reduced off-target editing (Fu et al., 2014). Truncated gRNAs (tru-gRNAs), as short as seventeen nucleotides, have been shown to destabilize the cleavage complex formation and reduce the time spent in the zipped conformation, allowing for more specific editing (Fu et al., 2014). However, it should be emphasized that manipulating the cleavage complex stability via truncated gRNAs is obtained at the expense of on-target activity (Pavel-Dinu et al., 2019) such that the balance between efficiency and specificity of genome editing should be carefully weighed. Furthermore, adding two extra guanine residues on the 5′ end of the spacer sequence (ggXX_20_ gRNA) had a variable effect on gene-editing performance in cultured cells, enhancing the guide specificity at specific genomic sites by significantly reducing off-target activity while maintaining the on-target efficiency (Cho et al., 2014). Nahar et al. demonstrated that introducing G-quadruplex (G4) structure at the 3′ end of the sgRNA resulted in increased *in vitro* serum stability and higher editing efficiency in the zebrafish embryos, compared to the unmodified sgRNA (Nahar et al., 2018). A much less pronounced effect was observed with G-rich hairpin at the 3′ end. On the other hand, G-rich hairpins or G4 structures at the 5′ end completely abolished Cas9-mediated cleavage (Nahar et al., 2018). A later study by Kocak et al. revealed that at off-target sites where RNA:DNA mispairing exists, and binding affinity is reduced, R-loop formation is hindered, while R-loop formation can commence normally at on-target sites (Kocak et al., 2019). In fact, it has been found that modifying the RNA secondary structure by engineering a hairpin onto the 5′ end of the sgRNA spacer sequence (hp-sgRNAs) significantly increases gene editing specificity in cells when complexed with various CRISPR effector nucleases (Kocak et al., 2019). In addition, the researchers achieved higher specificity using the engineered hairpin structures than with the tru-gRNA analog when tested side by side. However, the extended sgRNAs showed a tendency to undergo intracellular digestion back to the original size. To that end, a combination of the truncated or hairpin-modified sgRNAs in tandem with the previously discussed terminal chemical modifications could prevent hairpin removal by intrinsic intracellular nuclease activity, thus maximizing the editing capabilities of engineered sgRNAs. It is important to note that the hairpin structures' design must meet stringent constraints for thermodynamic stability since below a specific free energy cut-off, the nuclease activity is severely impaired. Interestingly, the hairpin structures had a strong negative effect on the *in vitro* nuclease activity due to the slower kinetics of the cleavage reaction. On the other hand, after sufficient time in cultured cells, the reduced cleavage rate proved beneficial for the overall specificity of the modified sgRNA-mediated editing.

### Partial DNA gRNA

It is well-documented that RNA residues in the crRNA and tracrRNA can be partially substituted for DNA residues without significantly impairing Cas9 activity both in *in vitro* cleavage assays and cultured cells (Rueda et al., 2017; Kartje et al., 2018; Yin et al., 2018; O'Reilly et al., 2019). The partial replacement of RNA nucleotides with DNA nucleotides in the crRNA has emerged as a potential approach to enhance CRISPR-Cas9 complex specificity by reducing off-target activity (Rueda et al., 2017; Kartje et al., 2018; Yin et al., 2018). The lower thermodynamic stability of the DNA-DNA duplex compared to the RNA-DNA duplex renders the partially DNA-substituted guide sequence of crRNA less tolerable to mismatches when interacting with genomic DNA. Kartje et al. demonstrated that *in vitro* cleavage of DNA duplexes by Cas9 could be facilitated by chimeric DNA-RNA crRNAs. Contrary to expectations, they showed that DNA substitutions inside the crRNA 3′ end, but not within the guide sequence, resulted in the Cas9-mediated cleavage being less tolerant of mismatches in the target sequence (Kartje et al., 2018). Conversely, Rueda et al. observed an increase in specificity in *in vitro* cleavage by replacing RNA residues with DNA residues inside of the guide sequence (Rueda et al., 2017). Yin et al. conducted a genome editing screen in Cas9 expressing HEK293T cells, which revealed that in living cells, the tail region, or the PAM-distal portion of the guide sequence was more amenable to DNA replacement than the seed region. They showed that replacing the ten RNA nucleotides in the PAM-distal region with DNA residues maintained on-target genome-editing activity (Yin et al., 2018). On the contrary, Cas9 endonuclease capability was severely impaired when crRNAs underwent substitutions inside the seed region. Incorporating more than twelve DNA nucleotides at the 5′ end or four DNA nucleotides at the 3′ end of the guide region was not tolerated (Yin et al., 2018). Hence, DNA-RNA hybrid crRNAs seem to present a plausible and cost-effective formulation for efficient and more accurate *in vitro* gene editing; however, it has yet to be validated in primary cells and animal models.

## Increasing the Safety of CRISPR-mediated Gene Editing by Curbing Cellular Toxicity and Immune Responses

CRISPR-Cas systems are bacterial mechanisms that researchers have worked determinedly to adapt to mammalian cells. However, as mentioned earlier, the CRISPR-Cas systems can evoke unwanted cellular and immune responses. Mammalian cells recognize the CRISPR complex as foreign and mount an immune response as a result (Cromer et al., 2018; Kim et al., 2018; Moon et al., 2019). Extensive research has been done on other nucleic acids therapies, such as siRNAs, mRNAs, and antisense oligodeoxynucleotides (ODNs) (Robbins et al., 2009; Burel et al., 2012; Kaczmarek et al., 2017; Meng and Lu, 2017) which can trigger immune responses; however, less is known about the immune recognition of gRNAs and the CRISPR system. Through a deeper understanding of the cause of the immune response, researchers have made strides to circumvent these deleterious side-effects by modifying the structure of the gRNAs.

### Removal of 5′ Triphosphate and Introduction of 2′-O-Me Uridine or Guanosine Residues

In human cells, foreign RNAs are recognized in the cytosol by pathogen-associated molecular pattern (PAMP) binding receptors, Retinoic acid-inducible gene 1 (RIG-1), also known as DExD/H-Box Helicase 58 (DDX58), and melanoma differentiation-associated gene 5 (MDA5). Upon encountering a PAMP motif on an RNA molecule, these proteins trigger a signaling cascade, eventually resulting in the upregulation of type 1 interferons and interferon-stimulated genes (Kell and Gale, 2015). Recently, in order to reduce the costs of producing a large amount of gRNAs, IVT by T7/SP6 phage RNA polymerases has become a popular method. However, since 5′-triphosphate (5′-ppp), which remains on the 5′-end of IVT RNA, is recognized as a PAMP, introducing IVT gRNA species into human cells can potentially trigger an innate immune response. Indeed, multiple research groups have reported cytotoxicity due to RNA-sensing, specifically via the RIG-1 pathway, and innate immune responses in human cells triggered by the 5′-triphosphate groups present on CRISPR gRNAs (Kim et al., 2018; Schubert et al., 2018; Wienert et al., 2018). Wienert et al., Kim et al., and Schubert et al. each examined various cell lines as well as different clinically relevant primary cells such as HSPCs, human peripheral blood monocytic cells (PBMCs), and CD4^+^ T cells. All cell types eventually exhibited a similar immune response to 5′-ppp gRNAs. Interestingly, the intracellular delivery method was deterministic in the immune response with nucleofection in HEK293 cells triggering a weaker and short-lasting type 1 interferon response, compared to lipofection (Wienert et al., 2018). Removal of the 5′-ppp groups by *in vitro* phosphatase treatment yielded 5′-hydroxyl gRNAs that could, in complex with Cas9 or Cas12a, achieve a high degree of mutagenesis in cell lines and primary human cells. This is actuated while triggering a reduced immune response similar to the synthesized gRNA species which are manufactured lacking 5′-ppp groups (Kim et al., 2018; Wienert et al., 2018). Furthermore, Schubert et al. demonstrated that the addition of 2′-O-Me and PS groups on the 2′-OH and phosphate backbone within synthesized gRNAs completely abolished any immune response (Schubert et al., 2018). This finding supported an earlier study that showed that the introduction of as few as two 2′-O-Me uridine or guanosine residues into either strand of a siRNA duplex eliminated any immune response (Judge et al., 2006). Hence, synthesized and chemically-modified gRNAs represent an optimal and clinically appropriate option for CRISPR-mediated gene editing in primary cells.

## Modifying gRNA to Increase HDR Efficiency

CRISPR-mediated DSBs can be repaired via the HDR pathway to allow for precise editing of DNA sequences, to correct genetic mutations, or to introduce novel genetic fragments. HDR uses a homologous DNA template, either endogenous (sister chromatid or homologous chromosomes) or exogenously introduced (donor template) sequences for genetic manipulation, and is, therefore, significantly less error-prone (Rouet et al., 1994; Porteus, 2016; Rodgers and McVey, 2016). By taking advantage of this endogenous repair pathway, efficient gene editing and gene knock-in are possible. Plasmid donors are problematic in clinical applications due to the risk of insertional mutagenesis and of triggering an immune response to foreign DNA. Therefore, Adeno Associated Virus (AAV) vectors have become a method of choice to introduce donor templates (Gaj et al., 2017). However, AAV vectors can also elicit immune responses, especially when used in primary cells or in human subjects, posing a critical caveat for gene therapy (High and Roncarolo, 2019). Therefore, to improve HDR efficiency and eliminate virus-induced immune responses, non-viral donor DNA delivery is crucial. In addition to engineering the Cas9 protein (Aird et al., 2018; Savic et al., 2018; Ling et al., 2020) or the DNA donor (Renaud et al., 2016) to improve HDR efficiency, modifications on the gRNA itself have great potential to enhance HDR efficiency in a non-viral manner to increase the relevance of the CRISPR-Cas9 gene-editing tool for many biotechnological applications.

### gRNA and Donor DNA Conjugates

**Figure 3:**
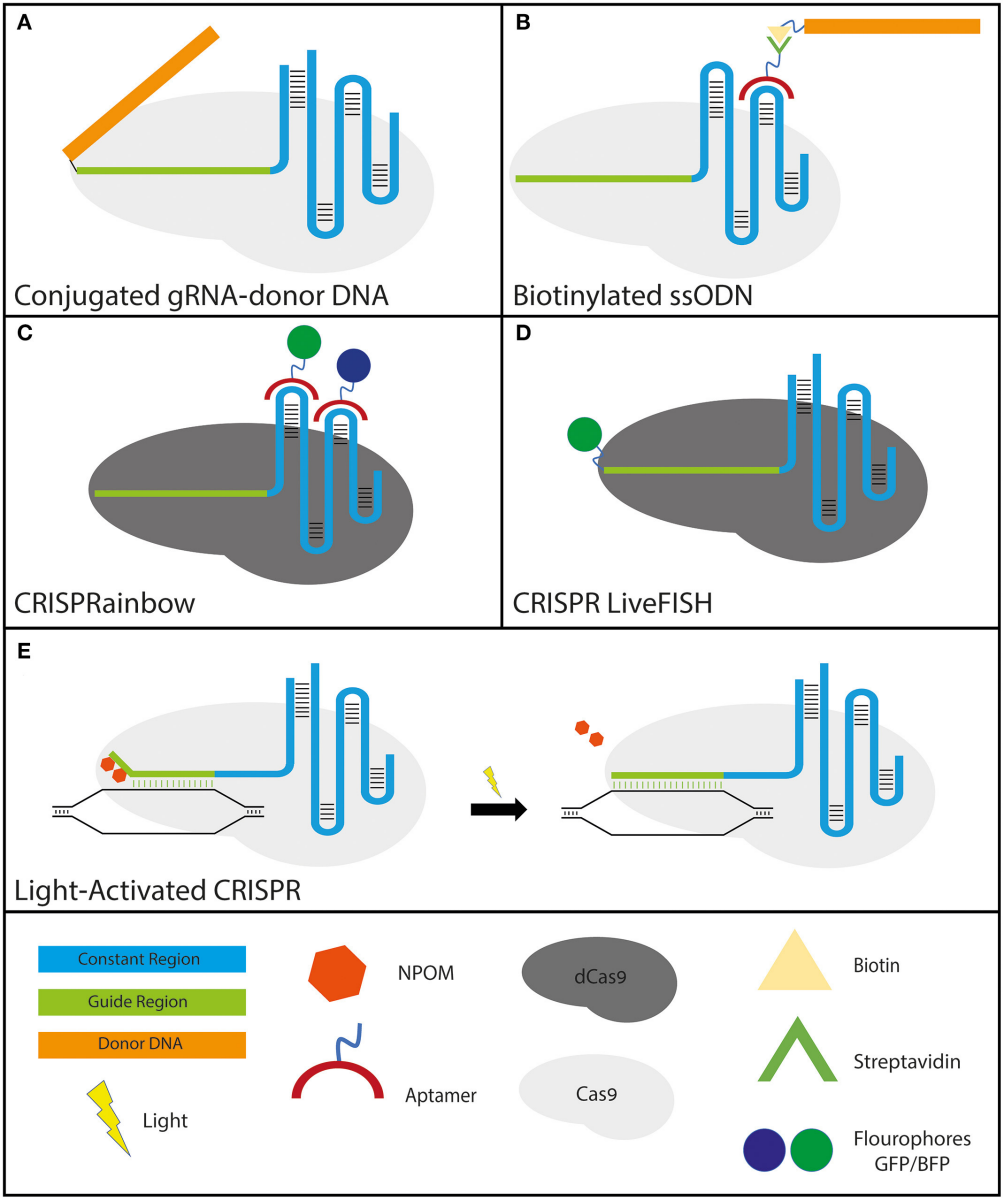
Various applications of engineered gRNAs. **(A,B)**–gRNA modifications to improve HDR: **(A)** crRNA-donor DNA conjugate. The donor DNA is fused to the 5′ end of the guide region. **(B)** sgRNA molecule with streptavidin-binding aptamers that attach to either the tetraloop or stem-loop 2 (the two loops protruding from the Cas9 molecule). The formulation has the donor ssODN bound to a biotin molecule that binds the streptavidin tightly to ensure the proximity of the donor DNA to the break site. **(C,D)**–gRNA modifications that utilize CRISPR-dCas9 specificity for high-resolution cellular imaging: **(C)** sgRNA molecule with fluorophore-bound aptamers binding to either the tetraloop or stem-loop 2 (for the same reason as mentioned above). GFP and BFP were shown solely as examples since CRISPRainbow covers the full spectrum of combinations. **(D)** CRISPR LiveFISH method utilizes crRNAs fused to a fluorophore at the 5′ end to actuate live intracellular staining without the need for cellular fixation. **(E)** Light-activated CRISPR to allow for control over synchronous editing across a cell population. Photocaging with light-sensitive 6′-nitropiperonyloxymethyl (NPOM) thymidine modifications on the distal portion of the guide region prevents the gRNA from binding completely to its DNA target. Following exposure to light, the NPOM modifications are released and complete binding and subsequent editing commence.

In order to improve the CRISPR-Cas9 system to actuate more efficient HDR two parameters must be improved upon: increasing the transfection efficiency of the DNA donor to the edited cells (Lee et al., 2017a) and localizing the DNA donor to the immediate vicinity of the DSB. To address these issues simultaneously, conjugated gRNA-donor DNAs, which ensures the proximity of the DNA donor to the cut site, have been engineered and have indeed showed improved HDR efficiency. These modified RNA-DNA hybrid molecules were engineered by conjugating an azide terminated DNA molecule with an alkyne modified crRNA. The engineered crRNA carrying the donor DNA was then annealed to standard tracrRNA and complexed with Cas9. The enhanced efficacy of the subsequent HDR showed that the conjugated gRNA could simultaneously act as a functional gRNA and donor DNA without the need for viral transduction (Lee et al., 2017b) (Figure 3A).

### Using RNA Aptamers on gRNA Backbone

Another approach that has been shown to increase HDR efficiency without the need to conjugate the gRNA to the donor DNA utilizes RNA aptamers. Adding RNA aptamers on either the tetraloop or stem-loop 2, which both protrude from the Cas9 protein, leaving them free of any interactions with the nuclease itself, are well-tolerated (Konermann et al., 2015). By exploiting these RNA aptamers, CRISPR-Display was established to introduce a targeted localization method to deploy large cargo, including protein-binding cassettes, to specific DNA loci (Shechner et al., 2015). Taking advantage of the strong natural interaction between streptavidin and biotin, it was shown that the addition of a streptavidin-binding RNA aptamer on the loop domains of the gRNA along with biotinylated single-stranded oligodeoxynucleotides (ssODNs) formed a highly effective tertiary complex (streptavidin-gRNA, biotin-ssODN, and Cas9). Using this tertiary complex they highlighted an improvement in both total HDR as well as in precise HDR efficiency (Carlson-Stevermer et al., 2017) (Figure 3B).

## Modifying gRNA to Utilize CRISPR-Cas9 as a Robust Method for Nuclear Imaging

Another application that modified gRNAs seek to improve upon is the existing imaging tools of chromosomal dynamics and genomic mapping, which are essential for comprehending a plethora of basic cellular nuclear processes. Previous attempts relied on the fusion of nuclease-deficient dead Cas9 (dCas9) with fluorescent proteins (Chen et al., 2013; Ma et al., 2015), which would be directed to the target loci by expressed sgRNAs. Furthermore, in order to improve the assay sensitivity by increasing sgRNA expression, Chen et al. modified the sgRNA sequence by conducting an A-U flip to remove a potential RNA PolIII terminator sequence, as well as extending a Cas9-binding hairpin structure (Chen et al., 2013). A different approach relied on simultaneously expressing engineered gRNAs containing MS2/PCP aptamers, MS2/PCP binding proteins fused to fluorescent proteins, and dCas9. This method provided efficient and reliable live-cell multicolor labeling of multiple chromosomal loci at the same time in live cells (Shao et al., 2016; Wang et al., 2016). By utilizing only one type of Cas protein (Cas9) and one type of gRNA, the systems developed by Shao et al. and Wang et al. allow greater simplicity, albeit limited to two colors unless applying additional dCas9 species fused to fluorescent proteins. The CRISPRainbow method further expanded the number of loci that can be viewed simultaneously by exploiting aptamer-carrying gRNA species (Ma et al., 2016) (Figure 3C). These modifications provide the CRISPR-Cas9 system the versatility to not only be used for genome editing but also for a deeper understanding of nuclear dynamics and mechanisms of action, including transcription, DNA replication, and DNA repair. In addition, the CRISPR LiveFISH method, with fluorophore-labeled gRNAs, presented a robust and novel approach using both dCas9 and dCas13 to enable real-time imaging of both DNA and RNA to track nuclear dynamics during genome editing and transcription in a wide range of live cells, including human primary cells (Wang et al., 2019) (Figure 3D).

## Modifying gRNA to Produce Inducible and Controlled Editing

Although tremendous progress in the quest to adapt the bacterial defense system to human cells has been made, much remains to be learned about the cellular response mechanisms and repair pathways in response to Cas-induced DSBs. Delivering CRISPR as an RNP complex is the most effective gene-editing method, but even then, cleavage is neither immediate nor synchronous across the treated cell population. This significantly hinders the ability to study the full spectrum of DSB formation and subsequent DNA repair dynamics. Extensive work has been done to produce inducible Cas9 systems to control nuclease activity by modifying the Cas9 protein to be activated only when induced chemically (Dow et al., 2015; Rose et al., 2017) or optically (Hemphill et al., 2015; Nihongaki et al., 2015a,b, 2017; Polstein and Gersbach, 2015; Richter et al., 2016; Zhou et al., 2018). However, a relatively simple and cost-effective method that allows optically-induced genome editing was recently demonstrated by adding photocaged light-sensitive 6′-nitropiperonyloxymethyl (NPOM) thymidine modifications on the distal portion of the gRNA (Liu et al., 2020; Moroz-Omori et al., 2020). Steric hindrance from these NPOM residues prevents binding of those residues, while the R-loop is successfully formed at the PAM-proximal residues. Due to the incomplete gRNA base pairing with the DNA site, the Cas9 remains catalytically inactive. Upon light stimulation (365 or 405 nm) that is not phototoxic to cells, as irradiation-induced damage is typically caused by wavelengths below 315 nm (Rastogi et al., 2010), photolysis of the NPOM moieties allows for complete gRNA base pairing, a conformational change in the Cas9 which in turn activates the nuclease domain, and DNA cleavage which is induced almost instantaneously. Indeed, significant DNA cleavage was generated within 30 seconds of light activation. This method of modifying the gRNA to facilitate light-induced Cas9 activation allows for synchronous DNA cleavage across a population of cells. This new CRISPR-Cas9 formulation is sure to lead to higher resolution, real-time DNA-repair analyses to better elucidate CRISPR-Cas9-induced DSB repair (Liu et al., 2020; Moroz-Omori et al., 2020) (Figure 3E).

## Conclusion

The FDA, EMA, and other oversight drug approval bodies implement rigorous and demanding tests before approving a given drug or therapy. Albeit, with CRISPR-mediated genome editing being a rapidly developing field, no standardized protocol for gRNA modifications has been generated yet for clinical studies, and every gRNA should be examined on an individual basis. Hence, our goal in this review article was to elucidate the entire repertoire of gRNA chemical modifications in order to allow the researchers in the field to make educated decisions while choosing the appropriate gRNA formulation that would fit the particular study design. Although there is a wide consensus regarding the profile of chemical modifications that improve the intracellular and intra-serum stability of guide RNAs, the proper design of the chemical gRNA modifications to improve the specificity of CRISPR-mediated genome editing is still to be determined. Notably, chemically-modified gRNAs are not restricted to the genome-editing via DSBs but can be exploited for a variety of applications involving catalytically-inactive Cas9 nucleases, Cas9 nickases, base editors and prime editors. (Anzalone et al., 2020). Though much more work remains to be done to optimize modified gRNAs for future routine human genome-editing-based therapies, there is no denying that the future of modified gRNAs and CRISPR-based therapeutics remains exceptionally bright. CRISPR-Cas systems, which can be engineered and modified with relative ease, provide a tremendous array of groundbreaking and versatile tools for programmable genome editing. The nucleic acid chemistry of gRNA enables expanding the array of nucleotide formulations from a native 4-letter RNA code to a wide range of phosphodiester, sugar ring, and nitrogen base modifications. In this review, we discussed the modifications on ribose ring and phosphodiester bonds, however, since it is well-known that RNA bases undergo a wide spectrum of modifications, such as 5-methylcytidine, or pseudouridine (Harcourt et al., 2017; Pan, 2018), which can ameliorate cellular immune responses (Hu et al., 2020), the potential to incorporate these could be a plausible future direction for engineering gRNAs. Certain modifications, such as the aforementioned MS and MSP modifications on the gRNA termini, are already being used worldwide as the quintessential standard for highly efficient genome editing. To that end, the first clinical trial, using C-C chemokine receptor type 5 (*CCR5*) knockout CD34^+^ HSPCs edited by gRNAs with the chemical modifications described in Hendel et al. (2015a), has already been conducted in an HIV-positive patient with Acute Lymphoblastic Leukemia (Xu et al., 2019). With additional clinical trials using CRISPR-Cas9 technologies commencing [Clinicaltrials.gov, #NCT03655678, and # NCT03745287, (Frangoul et al., 2020)] we expect synthetically modified gRNA-based therapeutics to take a major leap in the years to come. Through more extensive testing and development of different gRNA modifications aimed to increase efficiency, specificity, and safety, as well as new applications such as cell imaging and payload delivery to the DSB sites, we are confident that a wide array of therapeutic and biotechnological applications of the CRISPR-Cas technology will be accelerated for the benefit of human society.

## Author Contributions

DA, MR, and AH contributed to the conceptualization of the review and wrote the paper. All authors approved the submitted version.

## Conflict of Interest

The authors declare that the research was conducted in the absence of any commercial or financial relationships that could be construed as a potential conflict of interest.
